# Traditional medicine in Incahuasi: An ethnobotanical study

**DOI:** 10.12688/f1000research.138398.1

**Published:** 2023-08-31

**Authors:** Jorge Guillermo Morales Ramos, Darwin A. León-Figueroa, María Susana Picón Pérez, Marco Agustín Arbulú Ballesteros, Enrique Guillermo Llontop Ynga, Luis A. Coaguila Cusicanqui, Sofía Mariagracia Morales Ramírez, Carlos Alberto Chirinos Ríos

**Affiliations:** 1 Facultad de Medicina Humana, Universidad de San Martín de Porres, Chiclayo, 14012, Peru; 2 Escuela de Medicina Humana, Universidad Señor de Sipán, Chiclayo, 14012, Peru

**Keywords:** Traditional medicine, ethnobotany, systematic botany, medicinal plants

## Abstract

**Introduction:** Understanding the use of medicinal plants as herbal medicines is considered essential for the survival and continuity of humanity. Since ancient times, the origin and development of natural and traditional medicine have been intrinsically linked to humanity struggle for survival. Nowadays, ethnobotanical studies are employed as a tool for the preservation and conservation not only of taxonomic biodiversity but also of cultural biodiversity.

**Methodology:** A descriptive research with a quantitative, non-experimental cross-sectional design was carried out. The study was conducted in six Quechua-speaking communities in the district of Incahuasi (3,000 meters above sea level), selected for convenience considering factors such as altitude, accessibility, and proximity to the city. A questionnaire was administered to 32 residents from the communities, who shared their knowledge about medicinal plants, providing relevant information about them. The gender of the participants was considered because men and women use traditional medicine and the knowledge of them is transmitted from parents to children.

**Results:** During the study, a total of 46 medicinal species were recorded, belonging to 42 genera and 22 botanical families. The most representative medicinal families used by the informants of the communities were *Asteraceae* (30.4%) and *Lamiaceae* (15.2%). It is also worth mentioning the genera Salvia and Baccharis, with 3 and 2 species respectively, which are commonly used to treat various ailments and diseases.

**Conclusions:** Ethnobotanical information was collected on the medicinal plants used by the community members of the selected communities in Incahuasi, and the corresponding data were recorded. A total of 46 plants were collected, with the majority belonging to the *Asteraceae* and *Lamiaceae* families.

## Introduction

The knowledge of using medicinal plants as herbal remedies is considered an important factor for the survival and continuity of the human population, especially in the case of populations that, for various reasons, ^1^ find it difficult to access official healthcare services and therefore rely on medicinal plants. ^2^

Since the appearance of human beings on Earth, all cultures existing in a specific space-time dimension have always met their needs, such as food, healing common ailments in each place, clothing, food processing, shelter, among others. As a result, cultural evolution, and the evolution of knowledge about the natural environment have progressed hand in hand. ^3^

From an anthropological perspective, human life is intimately related to plant biodiversity and depends on it in various ways in which they are used, which is determined by cultural evolution in general. Therefore, regarding medicinal plants, the diversity in their utility and multiple applications can be studied transculturally, inferring changes in their use over time. ^4^

The origin of natural and traditional medicine is closely linked to that of humanity and mankind's struggle for survival. Traditional medicine, also known as unconventional medicine or complementary medicine, is a central component in healthcare systems worldwide. ^5^ The World Health Organization (WHO) defines traditional medicine as the sum of skills, knowledge, and practices used to maintain health, diagnose, prevent, improve, and treat physical and mental illnesses, based on beliefs, theories, and experiences specific to different cultures. ^6^

Over 80,000 out of the 250,000 species of flowering plants are used by human civilizations for medicinal purposes. ^7^ The use of Traditional Chinese Medicine (TCM), which dates back 3,000 years, has become popular worldwide, especially in China. However, Traditional Japanese Medicine, with a history of 1,500 years and including Kampo-yaku (herbal medicine), has also been incorporated. Nearly 148 different Kampo formulations can be prescribed within the Japanese national health insurance system. ^8^

Ethnobotany is a discipline that studies the interaction between humans and their plant environment, taking into account three dimensions: (1) cultural perception and taxonomy, (2) the biological and cultural dimension of plant use and management, and (3) cultural foundations and conservation of natural resources. ^9^ Ethnobotanical studies are used as a tool for conservation, not only of biological diversity but also of cultural heritage. ^10^ The ethnobotanical approach is one of the most specific and successful methods as it provides important clues for the discovery of new drugs or information about the pharmacological effects and biological impact of plants. This knowledge is rooted in accumulated practical knowledge and experiences over time by many ancient civilizations. ^11^^,^^12^

In general, ethnobotanical studies are one of the main steps in the identification and development of drugs from medicinal plants. ^13^

A clear example in the field of the influence of ethnobotany and the study of the medicinal properties of plants is the cure for malaria through the use of artemisinin, an active ingredient extracted from a Chinese plant called *Artemisia annua*, known as “qinghao” and “sweet wormwood”. This discovery earned Youyou Tu from the Republic of China the Nobel Prize in Medicine in 2015. ^14^^,^^15^

Various ethnobotanical studies have been conducted in the Amazon, where a diverse range of ethnic and cultural groups of indigenous and non-indigenous peoples depend on natural resources for their subsistence. One of these studies established an inventory with the objective of assessing the ethnobotanical knowledge of the inhabitants of three communities residing in the Marajó-Pará Coastal Island Marine Extractive Reserve, located at the mouth of the Amazon. The study was carried out using semi-structured interviews and participatory observation methods. Additionally, a non-probabilistic sampling through rational selection was employed. The study utilized indices such as total species diversity (SDtot) and informant diversity (IDs), consensus of use value (UDs), and use diversity value (UDs). The results allowed for the identification of a list of 215 ethnobotanical species, with 79 of them being cited by the interviewed inhabitants of the three communities. The most frequently cited category was medicinal plants. ^16^

Another research, conducted in 5 Shuar communities residing in the province of Zamora Chinchipe, in the Amazon region of Ecuador, collected information on the use of medicinal plants from 60 inhabitants over the age of 18. This study allowed for the determination of the significant use level (NUS), the use value index (IVU), and the ethnomedicinal knowledge index (ICE). The ICE revealed that it is concentrated among housewives, farmers, shamans or healers, and elderly individuals. The same study documented 69 species from 34 botanical families with medicinal uses. ^17^

In the Peruvian Amazon, an ethnomedicinal inventory was also conducted, analyzing its importance in 4 communities located on the outskirts of the San Antonio Private Conservation Area, in the province of Chachapoyas, Amazonas region. In the same study, the most frequently cited medical disorders among the residents (FCI), the most important medicinal plants in terms of relative importance (IR), and the level of cultural knowledge in each studied community (H index) were determined. A total of 124 medicinal species belonging to 104 genera and 47 taxonomic families were listed, with *Lamiaceae* and *Asteraceae* being the most representative families. ^18^

A systematic review of various ethnographic studies was carried out in Mexico, focusing only on species from the Verbenaceae family that were used as herbal resources. A total of 41 taxa corresponding to 12 genera and 37 species were recognized, with *Lantana camara, Verbena carolina, Phyla scaberrima,* and *Lippia graveolens* being the most cited ones. Furthermore, 92% of the species were used to treat digestive system disorders. ^19^

Another article highlights a systematic review on the ethnobotany, phytochemistry, and pharmacology of *Senecio tephrosioides Turz*, a plant from the Asteraceae family, which is a traditional medicinal plant used in ancient medicine systems in Peru and holds significant ethnopharmacological value. The plant contains various active compounds, such as terpenes (1-isopropyl-4-methyl bicyclo[3.1.0] hexane and terpinene), phenolic compounds (5,7-dihydroxy-4',6',8-trimethoxyflavanonone; 3',5-dihydroxy-4-methoxy-7-O-rhannoglucoside), flavonoids (quercetin-3-rutinoside), pyrrolizidine alkaloids (neoplatifiline, senecionine, seneciphylline, seneciphylline N-oxide, and senkirkine), and lactones (sylflavanonone and 5,4-dihydroxychalcone). ^20^

This research will be of great interest for further exploration of the beneficial effects of medicinal plants, and their inventory would help establish common characteristics among different cultures related to their use in treating diseases. Additionally, it is observed that if a plant species is common in a specific geographic area, its use is often specific to a particular community, symbolizing valuable knowledge that is being sought to protect and preserve. Such preliminary investigations, from a public health perspective, emphasize the relevance of traditional medicine, which should be understood as a community that formulates, interprets, participates, and addresses the health problems of its community. Moreover, it aims to contribute to a therapeutic option of natural origin based on traditional knowledge.

The objectives of this study are to document the ethnobotanical data of medicinal plants in Incahuasi and to understand the native medicinal flora present in the area.

## Methods

The study was conducted in the Peruvian district of Incahuasi, a Quechua word meaning “house of the Inca,” named after an ancient indigenous city located at an altitude of 3,100 meters above sea level (m.a.s.l.). It is one of the six districts of the Ferreñafe province, located in the Lambayeque region, Peru. Incahuasi is situated in the high Andean zone and consists of 127 populated centers (cps), ranging in elevation from 203 to 3,447 meters above sea level. The study focused on Quechua-speaking inhabitants who are users of traditional medicine, consumers, and herbalists from various populated centers in the district of Incahuasi. The cps were selected for convenience, taking into account factors such as altitude, accessibility, and proximity to the city.

The gender of the participants was considered, because men and women use traditional medicine and the knowledge of them is transmitted from parents to children. The selected cps were: Incahuasi, Sinchihual, Huasicaj, Tungula, Uyurpampa, and Huar-huar. All the collected plants originated from the Jalca (3,200 meters above sea level), were taxonomically identified. They were then organized systematically based on their medicinal use.

### Population and study sample

The study population consisted of informants from 6 populated centers in the district of Incahuasi, with an approximate total population of 3,200 residents. To determine the sample, 32 participants were selected, including 28 users of traditional medicine and 4 herbal healers, one of whom was a midwife.

A non-probability convenience sampling method was used to select the participants.

### Data collection

For data collection, the following activities were carried out: 1) Data collection began in January 2022 and concluded in December 2022, taking advantage of favorable weather conditions with lower precipitation. For the initial outing, a truck was hired to transport the project staff. This truck was used to survey the populated centers and conduct a pilot survey with some residents. 2) Transportation from the city of Chiclayo to Incahuasi (operations center) and vice versa was done using public transportation, with an approximate duration of 8 hours. Data collection and identification of medicinal plants were scheduled every 15 days, from Friday to Sunday. 3) The project leader, one of the co-authors, a botanist hired for on-site plant identification and systematization, and a Quechua-speaking translator were present on all outings. 4) The researchers traveled on foot to the selected populated centers to administer questionnaires to residents who met the inclusion criteria. They also visited healers and midwives living in rural areas. 5) When necessary, a Quechua-speaking translator was available to ask questions and translate the responses. A recorder was used as an additional tool for data collection, and the answers were recorded on the questionnaires. 6) The identification of medicinal plants was conducted on-site in the Jalca, located at an altitude of 3,200 meters above sea level, by the botanist who took photographs of the plants for taxonomy purposes ( https://zenodo.org/record/8231817). This process took place over the course of a year, considering the plants development and flowering stages. Finally, they were systematically organized based on their medicinal use. 7) It is important to mention that data collection was delayed during periods of high precipitation. While it was possible to reach the city, traveling to the populated centers during those times was not possible. The corresponding permits for the identification of the medicinal plants were processed through the competent Peruvian agency “Servicio Nacional Forestal y de Fauna Silvestre” (SERFOR).

After conducting the interviews, the data was transcribed using Microsoft Excel 2019 software. This allowed for the recording of responses provided by each participant in the study, with each record identified by a code to ensure the anonymity of each response. Additionally, the collection of medicinal plants included recording their order, family, species, and common name. The collected data was used to generate frequency tables and summary tables. To ensure data integrity, validation functions in Excel were employed to verify that all data accurately corresponded to each of the previously recorded medicinal plant names. After conducting the interviews, the data was transcribed using Microsoft Excel 2019 software. This allowed for the recording of responses provided by each participant in the study, with each record identified by a code to ensure the anonymity of each response. Additionally, the collection of medicinal plants included recording their order, family, species, and common name. The collected data was used to generate frequency tables and summary tables. To ensure data integrity, validation functions were employed in Excel to verify that all data precisely corresponded to each of the previously recorded medicinal plant names.

A questionnaire used was validated in a study carried out in 2016 by Gallegos *et al.* ^21^ called “Design and validation of the U-PlanMed questionnaire to identify the use of medicinal plants in Babahoyo, Ecuador” ( https://zenodo.org/record/8231817). The questionnaire was built with 6 domains, expressed in questions closed and multiple choice that were directed to identify the plants and parts used, therapeutic applications, purpose, forms of preparation, routes of administration, doses and duration of treatment. Cronbach's α values and even-odd reliability coefficient of the measuring instrument on the use of medicinal plants in rural communities was 0.82.

The questionnaire ^45^ consisted of 3 items related to sociodemographic aspects and 16 items on the use of medicinal plants, the parts of the plant, its preparation, the diseases that are treated with them, the doses, the form of administration and the duration of treatment.

### Data analysis

To obtain the results, the statistical software SPSS v. 27 and Excel 2019 were used to analyze the data. Simple frequency tables and bivariate tables were generated as tools for analysis.

### Selection criteria

***Inclusion criteria***

• Residents of both genders
• Quechua-speaking residents
• Residents older than 20 years
• Residents users of traditional medicine

***Exclusion criteria***

• Foreign residents settled in Incahuasi
• Users of conventional medicine

### Ethical considerations

The research complies with the ethical principles established in the Declaration of Helsinki, where autonomy and informed consent are fundamental elements of the scientific process. All research participants were informed in advance of the purpose of the study and freely agreed to participate by signing an informed consent letter that established the limits of their participation. The research was approved by the Ethics Committee of the Universidad de San Martín de Porres, Filial Norte, Chiclayo - Peru, through letter No. 139-2021 - CIEI-FMH-USMP, dated February 22, 2021.

## Results

Table 1 presents the classification of sociodemographic variables. It can be observed that the majority of the interviewed individuals are residents of the populated center of Incahuasi. Regarding the gender of the surveyed residents, there is no significant difference between both genders. Lastly, the age group with the highest frequency is the 50-59 age group, representing approximately 47% of the surveyed population.

**Table 1.** Sociodemographic factors, population centers, and frequency of interviewees.

Sociodemographic	Category	n	hi%
Population centers	Callima	2	6.3%
	Incahuasi	22	68.8%
	Laquipampa	2	6.3%
	Sinchiwal	2	6.3%
	Tungula	2	6.3%
	Wasikaq	2	6.3%
Gender	Female	15	46.9%
	Male	17	53.1%
Age range	20-29 years old	1	3.1%
	30-39 years old	4	12.5%
	40-49 years old	5	15.6%
	50-59 years old	15	46.9%
	> 60 years old	7	21.9%
	Total	32	100.0%

Table 2 displays the taxonomic classification of the identified medicinal plants, organized by class, order, family, species, and common name. A total of 46 species of medicinal plants were collected, belonging to 42 genera and 22 botanical families. The most representative botanical families, with a high percentage of species, were *Asteraceae* (30.4%) and *Lamiaceae* (15.2%). The genera *Salvia* (3 species) and *Baccharis* (2 species) also stand out.

**Table 2.** Taxonomic botany of medicinal plants used in the district of Incahuasi.

Type of plant	Order	Family	Species	Common Name
***Magnoliopsida***	*Lamiales*	*Lamiaceae*	*Salvia revoluta* Ruiz & Pav.	“Cutiquero"
			*Salvia oppositiflora* Ruiz & Pav.	“Chochogón”
			*Salvia ochrantha* Epling	“Jarua chochogón”
			*Minthostachys tomentosa* (Benth.) Epling.	“Chamagasa”, “Chancua”
			*Mellisa officinalis* L.	“Toronjil”
			*Minthostachys mollis* (Benth) Grieseb	“Muña”
			*Clinopodium taxifolium* (Kunth) Govaerts	“Romero de campo”
		*Verbenaceae*	*Duranta sp.*	“Garbanso”
			*Verbena sp*	“Verbena”
		*Plantaginaceae*	*Plantago major L.*	“Llandén”
	*Dipsacales*	*Viburnaceae*	*Sambucus peruviana* Kunth.	“Sauco”, “Sabuco”
	*Myricales*	*Myricaceae*	*Morella pubescens* (Humb. et Bonpl. ex Willd.) Wilbur	“Chicher", "Chichero”
	*Brassicales*	*Brassicaceae*	*Nasturtium officinale* W.T. Aiton.	“Vertius”
			*Matthiola incana* (L.) R.Br.	“Alhelí”
			*Brassica nigra* (L.) W.D.J.Koch	“Rabana”
	*Rosales*	*Urticaceae*	*Urtica urens L.*	“Changa”
		*Rosaceae*	*Margyricarpus pínnatus* (Lam.) Kuntze	“Perlilla”
	*Asterales*	*Asteraceae*	*Smallanthus microcephalus* (Hieron) H.Rob.	“Shita”
			*Ambrosia arborescens* Mill.	“Marco”
			*Paranephelius ovatus* Wedd.	“Pacha rosa”
			*Achyrocline alata* (Kunth) DC *.*	“Hashanco hishpincu”
			*Cichorium intybus* L *.*	“Achicoria”
			*Baccharis tricuneata* (L.f.) Pers.	“Pichana”
			*Baccharis latifolia* (Ruiz & Pav.) Pers.	“Chilca”
			*Cronquistianthus lavandulifolius* (DC.) R. M. King & H. Rob.	“Curbush” “Corbos”
			*Tagetes filifolia Lag.*	“Anís de monte”
			*Matricaria recutita L.*	“Manzanilla”
			*Jungia paniculata* (DC.) A.Gray	“Matico”
			*Schkuhria pinnata* (Lam.) Kuntze	“Canchalagua”
			*Hypochaeris sp.*	“Diente de león”
			*Ageratina exsertovenosa* (Klatt) R. King. & H. Rob.	“Becerro vario”
	*Piperales*	*Piperáceae*	*Peperomia inaequalifolia* Ruiz & Pav.	“Congona de monte”
	*Solanales*	*Solanaceae*	*Iochroma grandiflorum* Benth.	“Cushej” “Lushaj”
			*Cestrum sp.*	“Hierba santa”
	*Myrtales*	*Myrtaceae*	*Myrcianthes ferreyrae* (McVaugh) McVaugh	“Rumelonche” “Blanche” “Lanche”
			*Eucalyptus globulus* Labill.	“Eucalipto”
	*Fabiales*	*Fabiaceae*	*Otholobium mexicanum* (L.f.) J.W.Grimes	“Culén”
	*Fagales*	*Betulaceae*	*Alnus acuminata* Kunth	“Aliso”
	*Malphigiales*	*Hypericaceae*	*Hypericum silenoides* Juss.	“Canchilagua”
	*Ericales*	*Ericaceae*	*Pernettya prostrata* (Cav.) DC.	“Mullaca”
	*Scrophulariales*	*Scrophulariaceae*	*Alonsoa sp.*	“Mormor”
	*Geraniales*	*Geraniaceae*	*Geranium sp*	“Asolanjea”
	*Liliopsida*	*Cyperaceae*	*Eleocharis sp.*	“Chompe ojsha”
	*Polygonales*	*Polygonaceae*	*Muehlenbeckia tamnifolia* (Kunth) Meisn.	“Chumbiaura”
***Equisetopsida***	*Equisetales*	*Equisetaceae*	*Equisetum bogotense* Kunth.	“Culicaballo”, “Cola de caballo”
***Polypodiopsida***	*Polypodiales*	*Polypodiaceae*	*Adianthum sp.*	“Culantrillo”

Table 3 presents a list of medicinal plants and their traditional uses, including the plant part used and the method of preparation. It can be observed that some plants, such as “Cutiquero,” are used to treat up to 11 different types of diseases or symptoms. Furthermore, from the data, it can be inferred that leaves are the most commonly used plant part by the residents, followed by stems, flowers, and roots, in that order. The most common method of preparation is infusion, followed by decoction, ointment, extract, and cooking. Regarding the combination of plants to treat specific illnesses, “asolanjea,” “perlilla,” and “chompe ojsha” are used for the treatment of muscle strains and injuries.

**Table 3.** Traditional medicinal use, part of the plant and form of preparation of the plants collected in Incahuasi.

Used plant	Traditional medical use	Plant part used	Presentation form
Achicoria	Bronchitis	Leaves and root	Decoction
			Infusion
Alhelí	Bronchitis	Flowers	Decoction
Aliso	Flu	Leaves, root, and flowers	Infusion
Anís de monte	Colic	Leaves	Decoction
Asolanjea	Accompanied by Perlilla and Chompe Ojsha, drunk to treat muscular strain or internal disorder due to a hit.	Whole plant	Infusion
Becerro vario	Infection of any kind	Leaves	Decoction
Blanche (lanche)	Cold	Leaves	Ointment
Canchalagua	Heart	Leaves	Decoction
Canchilagua	Infection	Leaves	Infusion
Chamagasa (chancua)	Stomach ache, gas	Leaves and stem	Infusion
Chicher	Infection of any kind	Leaves	Decoction
	Flu	Leaves and flowers	Infusion
	Stomach	Leaves	Decoction
	Stomach ache	Leaves and flowers	Infusion
Chilca	Flu	Leaves	Decoction
Chochogón	Stomach ache	Leaves and stem	Decoction
	Expulsion of the placenta and stopping bleeding Altitude sickness, bad wind	Leaves and stem Leaves	Decoction Infusion
Chompe ojsha	Accompanied by Perlilla and Asolanjea, drunk to treat muscular strain or internal disorder due to a hit.	Whole plant	Infusion
Chumbiaura	Bronchitis	Leaves	Extract
Cola de caballo	Inflammation	Leaves	Infusion
Colandrello	For menstrual cramps in combination with oregano, every night, until the pain is gone	Whole plant	Infusion
Congona de monte	Bronchitis	Leaves	Ointment
Congonita	For nerves (Infusion)	Stem and leaves	Infusion
Corbos	Infection of any kind	Stem and leaves	Decoction
Culén	Stomach ache	Leaves	Cooking
	Cold	Leaves	Decoction
	Flu	Leaves	Infusion
Culi caballo	Inflammation	Leaves	Infusion
Curbush (corbos)	Colic (Chucaque)	Leaves, root and flowers	Infusion
	Stomach ache	Leaves	Decoction
Cushej (Luchaj)	Stomach ache	Leaves and flowers	Decoction
Cutiquero	Air, Flu	Leaves	Infusion
	Diarrhea	Stem and leaves	Decoction
	Headache	Stem and leaves	Decoction
	Stomach ache	Leaves	Infusion
	Stomach ache	Stem and leaves	Infusion
	Knee pain	Leaves	Infusion and Ointment
	Hemorrhage	Stem and leaves	Infusion
	Bad air	Stem and leaves	Extract
			Infusion
	Flu	Stem and leaves	Decoction
			Infusion
	Mountain sickness	Stem and leaves	Ointment
	Anti-abortion	Stem and leaves	Infusion
Diente de león	Infection of any kind	Whole plant	Decoction
Eucalipto	Flu	Leaves	Decoction
			Infusion
		Leaves, root and flowers	Infusion
Garbanzo	Stomach ache	Leaves	Infusion
Hashanko hishpinku	Earache	Leaves	Extract
Hierba santa	For Fever (rub and drink)	Leaves	Infusion
Jarua chochogon	Expulsion of the placenta and stop bleeding	Stem and leaves	Decoction
Lanchi	Flu, throat, “time water”	Leaves	Infusion
Llanden (Llantén)	Urinary infection	Leaves	Infusion
	Anti-inflammatory, Flu	Leaves	Decoction
	Liver	Leaves	Decoction
Locha (luchaj, loqcha)	Stomach ache	Leaves	Infusion
	Bad air or “Mal de aire”	Leaves	Infusion
	Expulsion of the placenta and stop bleeding	Leaves	Decoction
Manzanilla	Colic	Leaves and flowers	Infusion
Marco	Chucaque	Leaves, root and flowers	Infusion
	Flu	Stem and leaves	Decoction
Matico	Flu	Leaves	Infusion
Matico de la sierra	Expulsion of the placenta and stop bleeding	Leaves	Decoction
Mor-mor	Stomach ache	Stem and leaves	Infusion
Mullaca	Expulsion of the placenta and stop bleeding	Leaves	Decoction
Muña	Stomach ache	Leaves	Decoction
	Colic	Leaves	Infusion
			Ointment
	Flu	Leaves	Extract
	Flu, Cold	Leaves	Infusion
		Leaves, root and flowers	Infusion
Muña, chamagasa	Stomach ache	Leaves	Infusion
Pacha rosa	Bronchitis	Leaves and flowers	Infusion
Perlilla	These 3 herbs are drunk when the person suffers muscle strain or internal disorder due to a hit.	Stem and leaves	Infusion
Pichana	Healing of wounds	Leaves, stem and flowers	Infusion
	Flu	Leaves	Decoction
Rabanas	Fever	Leaves	Decoction
Romero	Stomach ache	Leaves and flowers	Infusion
	Cold	Leaves and flowers	Infusion
	Flu	Leaves and flowers	Decoction
Rumelonche	Cold	Stem and leaves	Decoction
Sauco	Expulsion of the placenta and stop bleeding	Leaves and flowers	Decoction
	Stomach ache	Leaves and flowers	Decoction
	Fever, Throat	Leaves and flowers	Decoction
	Flu	Leaves and flowers	Decoction
		Leaves and flowers	Decoction
Shita	Flu	Leaves	Infusion
Verbena	Stomach ache	Stem and leaves	Infusion
Vertius	Headache	Leaves	Infusion
Yurajlucha (lushaj)	Bad air or “Mal de aire”	Leaves	Infusion

Table 4 provides a detailed description of diseases and the plants used to treat them. It also gives an overview of the number of plants used to treat a single disease or multiple diseases. For example, 24 plant species are used to treat acute respiratory infections, while 14 species are employed for treating gastroenterocolitis. Overall, the information extracted from the table is valuable for understanding the use of medicinal plants in various treatments.

**Table 4.** Diseases and medicinal plant used in the treatment.

Diseases	Plant used
Otitis	1. Hashanko, hishpinku
Menstrual cramps	1. Culantrillo
Generalized infection	1. Becerro varia /2. Corbos/Curbush 3. Diente de león /4. Checher/Chicher rojo/Chichero
Acute respiratory infections	1. Aliso /2. Marco /3. Chilca /4. Pichana /5. Rabanas /6. Hierba santa /7. Achicoria 8. Romero /9. Eucalipto /10. Luchaj /11. Matico/Matico de la sierra /12. Alelí /13. Muña, Chamagasa, chancua /14. Checher/Chicher rojo/Chichero /15. Chumbiaura /16. Lanchi, Blanche /17. Rumelonche /18. Culén /19. Pacha rosa /20. Congona de monte, Congonita /21. Llanden, Llantén /22. Cutiquero amarillo /23. Sabuco, Cebunka, sauco 24. Shita
Muscle contraction	1. Marco /2. Cosbos/Curbush
Localized infections	1. Pichana
Myopathies	1. Chompe ojsha /2. Asolanjea /3. Perlilla
Urinary infections	1. Cola de caballo/Culi caballo
Microbial infections	1. Canchilagua
Childbirth	1. luchaj /2. Matico/Matico de la sierra /3. Jarva /4. Chochogón /4. Sauco
Gastritis	1. Muña, Chamagasa, chancua
Gastroenterocolitis	1. Muña, Chamagasa, chancua /2. Chicher /3. Chuchugón, 4. Cutiquero /5. Sauco /6. Anís /7. Verbena /8. Mor – mor /9. Romero /10. Curbush /11. Garbanzo /12. Locha (Luchaj /13. Cushej /14. Manzanilla
Gastroenteritis	1. Checher/Chicher rojo/Chichero 2. Culén
Tension headache	1. Vertius 2. Cutiquero, Cutiquero amarillo
Nervous disease	1. Congona de monte, Congonita
Urinary infection	1. Llanden, Llantén
Hepatic inflammation	1. Llanden, Llantén
Vertigo	1. Chuchugón /2. Cutiquero
Uterine atony	1. Cutiquero, Cutiquero amarillo
Coronary disease	1. Canchalagua

Table 5 highlights the most commonly treated diseases using medicinal plants, with viral infections being the highest at 25.4%. Additionally, certain symptoms are also addressed, such as stomach pain at 16.6%, placenta expulsion and reduction of bleeding at 5.8%, “mal de aire” (a condition related to cold or sudden temperature changes) at 5.7%, altitude sickness (soroche) at 4.0%, headaches at 2.9%, muscle strains at 2.9%, and inflammation at 2.9%.

**Table 5.** Frequency and percentage of traditional medicinal use of plants in Incahuasi.

Traditional medicine use	Frequency	Percentage
Antiabortion	1	1.0%
Pneumonia	6	5.9%
Chucaque	2	1.9%
Colic	2	1.9%
Heart	1	1.0%
Healing of wounds	1	1.0%
Anti-inflammatory	3	1.8%
Diarrhea	2	1.9%
Headache	3	2.9%
Stomach ache	17	16.6%
Knee pain	1	1.0%
Gas	1	1.0%
Earache	1	1.0%
Muscle strain or internal disorder due to a hit	3	2.8%
Expulsion of placenta and stopping bleeding	6	5.8%
Fever	4	3.9%
Cold	8	7.8%
Gastritis	2	1.7%
Flu	11	10.7%
Hemorrhage	1	1.0%
Liver	1	1.0%
Infection of any kind	6	5.9%
Bad air or “Mal de aire”	6	5.7%
Menstrual cramps	1	1.0%
For nerves (infusion)	1	1.0%
Sore throat	9	8.8%
Kidney	1	1.0%
Altitude sickness (“soroche”)	2	3.0%
Total	103	100.0%

## Discussion

The results presented in Table 1, considering sociodemographic factors, indicate that both genders significantly utilize and practice traditional medicine, knowledge that has been passed down from ancestors to their children. ^22^ The vast majority of residents (98%) first consult herbal healers and then seek medical assistance at health centers. Accordingly, highland populated centers embrace traditional medicine, biomedicine, self-treatment, and other options based on their own worldview. ^23^ The study suggests that there is no significant difference between both sexes, and the age range with the highest frequency is between 50 to 59 years, representing approximately 47% of the surveyed population. This finding aligns with the data showing that the ethnomedicinal knowledge is concentrated among elderly individuals, healers, or shamans. ^24^

The results extracted from Table 2 indicate that the most representative families are *Asteraceae* and *Lamiaceae*, with the leaf being the most commonly used part ( Table 3), which coincides with published works. ^25^^‐^^27^ Asteraceae is the most diverse group of flowering plants on the planet, with records of ethnobotanical uses dating back to ancient times. A study published in 2021 established that Asteraceae species found in Quinua (Ayacucho) and Lircay (Huancavelica) were used for digestive and genitourinary ailments (22.8% and 13.1% respectively). ^28^ 80.0% of the Asteraceae collected in the populated centers of Incahuasi are used for the treatment of infections (earache, stomachache, bronchitis, cold). The most representative collected Asteraceae species are: Ambrosia arborescens Mill. “Marco,” and two species from the genus Baccharis: *Baccharis tricuneata* (L.f.) *Pers.* (pichana) and *Baccharis latifolia* (Ruiz & Pav.) Pers.

*Ambrosia arborescens Mill.* 'Marco' is used in traditional Incahuasi medicine for the treatment of muscular contracture, cold, and chucaque. A phytochemical study identified 113 compounds, including β-acoradiene (15.3%) and chrysanthenone (11.3%), as the main constituents of the essential oil with antioxidant capacity. ^29^ It also exhibits anti-inflammatory activity, with metabolites such as tannins and flavonoids being identified. A systematic review refers to the use of 'Marco' for treating the common cold. ^30^

*Baccharis tricuneata* (L.f.) *Pers.* (pichana) and *Baccharis latifolia* (Ruiz & Pav.) *Pers.* (chilca): both species are used by the interviewees for the treatment of the common cold. However, a study published in 2023 states that both species are indicated as antibacterial and antidiabetic agents. ^31^^,^^32^ In general, the genus is used as an anti-inflammatory due to the presence of ethyl acetate and hexane fractions. ^33^

Among the most representative species of the Lamiaceae family, we find *Salvia revoluta Ruiz & Pav.* “Cutiquero,” which is used by the residents of Incahuasi for the treatment of bad air, influenza, common cold, diarrhea, headache, stomach pain, knee pain, and bleeding during childbirth; and *Minthostachys mollis* (Benth) Grieseb “muña.” The largest genus within the *Lamiaceae* family is Salvia, which encompasses 1,010 species, several of which are globally used in traditional medicine for the treatment of conditions such as angiogenesis, viral and bacterial infections, inflammation, and oxidative stress. ^34^

*Salvia revoluta Ruiz & Pav.* “Cutiquero.” Central and South America host the highest number of Salvia species worldwide, with 510 species. ^35^ A study reveals the antibacterial power of the genus in 11 species in Iran, where the main components detected were β-caryophyllene, 1,8-cineole, and α and β-pinene. The most susceptible organisms were Salmonella typhi and Bacillus subtilis. ^36^

*Minthostachys mollis* (Benth) Grieseb., known as “muña,” is commonly used in traditional South American medicine. ^37^ In the Incahuasi community, it is used as an infusion to treat various health disorders such as stomach cramps, flu, and colds ( Tables 3, 4, 5). Peruvian muña produces compounds of medicinal interest, and one of the most abundant compounds in muña oil is the monoterpene Pulegone. Pulegone possesses anti-inflammatory activity as it inhibits the expression of lipopolysaccharides and regulates inflammation in vitro by modulating the inducible nitric oxide synthase (iNOS) isoform and cyclooxygenase-2 (COX-2) expression. It participates in the inhibition of NF-κB (nuclear factor kappa B) and MAPKs (nitrogen-activated protein kinases) signaling pathways, while strengthening the Nrf-2/HO-1 (nuclear factor E2-related factor 2/heme oxygenase 1) pathways. In vivo, it suppresses NLRP3 inflammasome and reduces cytokine production. Additionally, it exhibits psychoactive activity and acts as an analgesic, antibacterial, antifungal, and insecticidal agent. ^38^ Nrf-2 plays a fundamental role in redox homeostasis. ^39^ Moreover, a cytoprotective effect has been demonstrated in gastric lesions induced in rats. ^40^

The majority of medicinal plant species in the Quero district are used for specific traumas, particularly for injuries and wounds. ^41^ In Chachapoyas, Peru, they are used as medical indications for digestive diseases. ^41^^,^^43^^,^^44^ However, this study shows that there is a higher number of plant species used for acute respiratory diseases and gastroenterocolitis ( Tables 4 and 5), which frequently include viral infections and the characteristic symptom of stomach pain. The research findings are consistent with the study conducted by Mostacero *et al.* ^45^

## Conclusion

During the study, ethnobotanical data on medicinal plants used in the selected communities of Incahuasi were recorded. Various aspects were collected, including the common names of the collected plants, their mode of use, the characteristics of the environment where they were found, whether they were used fresh or dried, the parts utilized, the preparation method of home remedies, as well as the form and frequency of administration. Additionally, the transmission of ancestral knowledge related to these medicinal plants was taken into account.

During the study, 46 medicinal plants were collected, with the majority of them belonging to the Asteraceae and Lamiaceae families, which were considered the most representative. Among the plants belonging to the Asteraceae family, Ambrosia arborescens Mill., known as “Marco,” *Baccharis tricuneata* (L.f.) *Pers.*, called “pichana,” and *Baccharis latifolia* (Ruiz & Pav.) Pers. were found. On the other hand, among the plants belonging to the *Lamiaceae* family, *Salvia revoluta Ruiz & Pav.*, known as “Cutiquero,” and *Minthostachys mollis* (Benth) Grieseb., known as “muña,” stood out.

### Limitations

The research results were obtained throughout the different seasons of the year. It should be noted that the climate posed a limitation for sample collection during the rainy season, while the altitude and rugged nature of the areas also presented a challenge. It is important to highlight that these results may vary over time and with the inclusion of a greater number of communities in the study. Additionally, since the interviewed population speaks Quechua, the presence of a translator was often required to facilitate communication.

## Data Availability

Zenodo: Traditional medicine in Incahuasi: ethnobotanical study. https://zenodo.org/record/8231817. ^46^ The project contains the following underlying data:
• Base de datos agrupada - General.xlsx Zenodo: Traditional medicine in Incahuasi: ethnobotanical study. https://zenodo.org/record/8231817. ^46^ This project contains the following extended data:
• Questionnaire The data are available under the terms of the Creative Commons Attribution 4.0 International license (CC-BY 4.0).
