# Supplementary material for: Traditional medicine in Incahuasi: An ethnobotanical study
Source: F1000Res. 2024 Apr 23;12:1075. Originally published 2023 Aug 31. [Version 2] doi: 10.12688/f1000research.138398.2 (PMC10993006; doi:10.12688/f1000research.138398.2)
Supplement: Peer review history

# Reviewer report: Reviewer response for version 2

**DOI:** 10.5256/f1000research.164103.r268960

**Published:** 2024-05-07

**Authors:** Adriana Beatriz Sánchez-Urdaneta

**Affiliations:** 1 Universidad Tecnica de Manabi, Portoviejo, Manabí Province, Ecuador

After reviewing the comments made to the manuscript “Traditional medicine in Incahuasi: An ethnobotanical study, it can be seen that the authors accepted the observations made, therefore, as far as I am concerned, I approve of the changes made and that they contributed to improve the quality of the manuscript.

Is the work clearly and accurately presented and does it cite the current literature?

Yes

If applicable, is the statistical analysis and its interpretation appropriate?

Yes

Are all the source data underlying the results available to ensure full reproducibility?

Yes

Is the study design appropriate and is the work technically sound?

Yes

Are the conclusions drawn adequately supported by the results?

Yes

Are sufficient details of methods and analysis provided to allow replication by others?

Yes

Reviewer Expertise:

My areas of expertise are plant morphoanatomy, plant physiology, crop production and management, some aspects of plant systematics.

I confirm that I have read this submission and believe that I have an appropriate level of expertise to confirm that it is of an acceptable scientific standard.

# Reviewer report: Reviewer response for version 1

**DOI:** 10.5256/f1000research.151588.r203201

**Published:** 2024-04-09

**Authors:** José Yovera Saldarriaga

**Affiliations:** 1 Universidad Nacional Santiago Antunez de Mayolo, Huaraz, Ancash, Peru

The study highlights the importance of knowing medicinal plants in populations with difficult access to conventional health services. It presents an Anthropological and cultural approach by connecting knowledge of the natural environment with human survival in the relationship between human life and plant biodiversity.

 It provides clarity and institutional support when defining traditional medicine; geographically expands the concept by mentioning examples in other latitudes, contributing to the cultural diversity of the investigation. Thus, it highlights the importance of ethnobotany in the conservation of biological biodiversity and cultural heritage.

 Methodologically, the study is well structured, it also considers ethical aspects and points out limitations, strengthening the integrity of the research approach.

 The results provide a comprehensive look at the use of medicinal plants in Incahuasi, highlighting their diversity, common uses and their role in the treatment of dissimilar diseases and symptoms; They accentuate the richness of traditional medicine in Incahuasi, the value of certain families and species in treating various conditions, highlighting their potential in local medical care.

Is the work clearly and accurately presented and does it cite the current literature?

Yes

If applicable, is the statistical analysis and its interpretation appropriate?

Yes

Are all the source data underlying the results available to ensure full reproducibility?

Yes

Is the study design appropriate and is the work technically sound?

Yes

Are the conclusions drawn adequately supported by the results?

Yes

Are sufficient details of methods and analysis provided to allow replication by others?

Yes

Reviewer Expertise:

My research area is cultural anthropology.

I confirm that I have read this submission and believe that I have an appropriate level of expertise to confirm that it is of an acceptable scientific standard.

# Reviewer report: Reviewer response for version 1

**DOI:** 10.5256/f1000research.151588.r224511

**Published:** 2024-04-03

**Authors:** José Mostacero León

**Affiliations:** 1 Facultad de Ciencias Biológicas, Universidad Nacional de Trujillo, Trujillo, Peru

The study highlights the importance of knowing medicinal plants in populations with difficult access to conventional health services. It presents an Anthropological and cultural approach by connecting knowledge of the natural environment with human survival in the relationship between human life and plant biodiversity.

 Provides clarity and institutional support when defining traditional medicine; geographically expands the concept by mentioning examples in other latitudes, contributing to the cultural diversity of the investigation. Thus, it highlights the importance of ethnobotany in the conservation of biological biodiversity and cultural heritage.

 Methodologically, the study is well structured, it also considers ethical aspects and points out limitations, strengthening the integrity of the research approach.

 The results provide a comprehensive look at the use of medicinal plants in Incahuasi, highlighting their diversity, common uses and their role in the treatment of dissimilar diseases and symptoms; They accentuate the richness of traditional medicine in Incahuasi, the value of certain families and species in treating various conditions, highlighting their potential in local medical care.

Is the work clearly and accurately presented and does it cite the current literature?

Yes

If applicable, is the statistical analysis and its interpretation appropriate?

Yes

Are all the source data underlying the results available to ensure full reproducibility?

Yes

Is the study design appropriate and is the work technically sound?

Yes

Are the conclusions drawn adequately supported by the results?

Yes

Are sufficient details of methods and analysis provided to allow replication by others?

Yes

Reviewer Expertise:

cultural anthropology

I confirm that I have read this submission and believe that I have an appropriate level of expertise to confirm that it is of an acceptable scientific standard.

# Reviewer report: Reviewer response for version 1

**DOI:** 10.5256/f1000research.151588.r224508

**Published:** 2024-03-18

**Authors:** César Grandez Ríos

**Affiliations:** 1 Departamento académico de botánica. Facultad de Ciencias Biológicas, National University of the Peruvian Amazon, Iquitos, Loreto, Peru

I have reviewed the article proposed by Morales Ramos JG, et al.

 I have detailed each section in order for the authors to carefully consider the observations and comments that need to be addressed and resolved. This division will facilitate a more effective review and allow for an appropriate response to each point made, thus contributing to the overall improvement of the content.

 Introduction:

 A thorough review has been carried out, incorporating references relevant to the proposed topic. As a result, the quality of both the writing and the information is considered adequate and compliant with established standards.

 Methodology:

 The methodology described fits consistently with the proposed research design, providing a detailed approach. It is notable that the study includes a data validation sheet, highlighting the rigor in data collection and analysis.

 Additionally, I suggest including mention of the herbarium, where the processing of the plants for identification and taxonomic classification was carried out. This additional information will not only enrich the transparency of the study but will also allow readers to better understand the source and context of the botanical data analyzed.

 Results

 The wording of the tables demonstrates an adequate level of accuracy. However, I propose to avoid repeating the full scientific names of the plants in order to follow appropriate standards. 

 Discussion 

 This section was written in detail. A comparison of the results with other relevant publications in the field was made. Furthermore, it has been ensured that references are up to date and relevant, thus contributing to the robustness and relevance of the information presented.

 Conclusions

 The conclusions are in line with the research objectives.

 References

 They comply with the Vancouver standards established in the journal.

Is the work clearly and accurately presented and does it cite the current literature?

Yes

If applicable, is the statistical analysis and its interpretation appropriate?

Yes

Are all the source data underlying the results available to ensure full reproducibility?

Yes

Is the study design appropriate and is the work technically sound?

Yes

Are the conclusions drawn adequately supported by the results?

Yes

Are sufficient details of methods and analysis provided to allow replication by others?

Yes

Reviewer Expertise:

Taxonomist, Ethnobotanist,  economic botanic

I confirm that I have read this submission and believe that I have an appropriate level of expertise to confirm that it is of an acceptable scientific standard.

# Response

**Published:** 2024-03-21

**Authors:** Jorge Guillermo Morales Ramos

**Affiliations:** Lambayeque, Universidad De San Martin de Porres - Filial Norte, Chiclayo, Lambayeque, Peru

**Reviewer 2: César Grandez Ríos**

**The reviewer says:**  I have reviewed the article proposed by Morales Ramos JG, et al. I have detailed each section in order for the authors to carefully consider the observations and comments that need to be addressed and resolved. This division will facilitate a more effective review and allow for an appropriate response to each point made, thus contributing to the overall improvement of the content.

**Our answer**: Dear Doctor, we are deeply grateful for the review you have done, which enhances our work. 

**The reviewer says:** Introduction: A thorough review has been carried out, incorporating references relevant to the proposed topic. As a result, the quality of both the writing and the information is considered adequate and compliant with established standards.

**Our answer**: Thank you for your comment.

**The reviewer says:** Methodology: The methodology described fits consistently with the proposed research design, providing a detailed approach. It is notable that the study includes a data validation sheet, highlighting the rigor in data collection and analysis.

**Our answer**: We agree with your assessment. 

**The reviewer says:** Additionally, I suggest including mention of the herbarium, where the processing of the plants for identification and taxonomic classification was carried out. This additional information will not only enrich the transparency of the study but will also allow readers to better understand the source and context of the botanical data analyzed.

**Our answer**: We have included the name of the herbarium.

**The reviewer says:** Results: The wording of the tables demonstrates an adequate level of accuracy. However, I propose to avoid repeating the full scientific names of the plants in order to follow appropriate standards.

**Our answer**: Your recommendation was taken into account.

**The reviewer says:** Discussion: This section was written in detail. A comparison of the results with other relevant publications in the field was made. Furthermore, it has been ensured that references are up to date and relevant, thus contributing to the robustness and relevance of the information presented.

**Our answer**: Thank you very much for your comments.

**The reviewer says:** Conclusions: The conclusions are in line with the research objectives.

**Our answer**: Thank you very much for your comments.

**The reviewer says:** References: They comply with the Vancouver standards established in the journal.

**Our answer**: Thank you very much for your comments.

# Reviewer report: Reviewer response for version 1

**DOI:** 10.5256/f1000research.151588.r224505

**Published:** 2024-01-10

**Authors:** Adriana Beatriz Sánchez-Urdaneta

**Affiliations:** 1 Universidad Tecnica de Manabi, Portoviejo, Manabí Province, Ecuador

Based on a detailed reading of the article, here is my analysis:

 The article has a logical and coherent structure. The introduction adequately presents the topic and its relevance. The methodology clearly describes the research design, study population, instruments used to collect data, and the analysis performed. The results are presented in an organized manner in tables that facilitate understanding. The discussion appropriately analyzes the findings, comparing them with previous studies cited in the references. Finally, the conclusions summarize the main findings of the study. There is an argumentative line that connects the different sections of the article. The objectives set out in the introduction are addressed throughout the document. The article includes an adequate number of quality bibliographic references that support the statements made. The references mostly follow the Vancouver style standards. Some details to improve: the volume number is missing in some journals, sometimes the page range of the cited articles is not included. Most references are current, after 2015, although some older relevant sources are also cited.

 The writing is generally clear and understandable, with good language quality. Tables and figures are mentioned in the text and add value by presenting results. The article complies with the ethical principles of research in human beings.

 However, there are some recommendations that I would like to make, with the aim of improving the manuscript. The names of families, orders and types of plants do not have to be written in italics. Numbers greater than 10 should be written in letters and not in numbers, unless they are preceded by a unit of measurement recognized by the international system. With respect to scientific names, once the genus is named, the following times it appears, the initial of the genus is used, unless a paragraph begins with a scientific name.

 For example: Baccharis tricuneata (L.f.) Pers. (pichana) and Baccharis latifolia (Ruiz & Pav.) Pers. (chilca), should be changed to Baccharis tricuneata (L.f.) Pers. (pichana) and B. latifolia (Ruiz & Pav.) Pers. (chilca), among other examples.

 In conclusion, the article has a very good quality in terms of structure, internal coherence, bibliographic support and writing. With some minor adjustments in the reference format, the document could be ready to be indexed.

Is the work clearly and accurately presented and does it cite the current literature?

Yes

If applicable, is the statistical analysis and its interpretation appropriate?

Yes

Are all the source data underlying the results available to ensure full reproducibility?

Yes

Is the study design appropriate and is the work technically sound?

Yes

Are the conclusions drawn adequately supported by the results?

Yes

Are sufficient details of methods and analysis provided to allow replication by others?

Yes

Reviewer Expertise:

My areas of expertise are plant morphoanatomy, plant physiology, crop production and management, some aspects of plant systematics.

I confirm that I have read this submission and believe that I have an appropriate level of expertise to confirm that it is of an acceptable scientific standard, however I have significant reservations, as outlined above.

# Response

**Published:** 2024-03-21

**Authors:** Jorge Guillermo Morales Ramos

**Affiliations:** Lambayeque, Universidad De San Martin de Porres - Filial Norte, Chiclayo, Lambayeque, Peru

**Reviewer 1: Adriana Beatriz Sánchez-Urdaneta**

 Thank you very much, dear doctor, for reviewing our article. Your comments and recommendations have been beneficial for improving the quality of the article. The comments raised are listed below. 

**The reviewer says:** Based on a detailed reading of the article, here is my analysis: The article has a logical and coherent structure. The introduction adequately presents the topic and its relevance. The methodology clearly describes the research design, study population, instruments used to collect data, and the analysis performed. The results are presented in an organized manner in tables that facilitate understanding. The discussion appropriately analyzes the findings, comparing them with previous studies cited in the references. Finally, the conclusions summarize the main findings of the study. There is an argumentative line that connects the different sections of the article. The objectives set out in the introduction are addressed throughout the document. The article includes an adequate number of quality bibliographic references that support the statements made.

**Our answer**: Thank you for your appreciation of the structure and wording of our article.

**The reviewer says:** The references mostly follow the Vancouver style standards. Some details to improve: the volume number is missing in some journals, sometimes the page range of the cited articles is not included. Most references are current, after 2015, although some older relevant sources are also cited.

**Our answer**: We have sequentially adapted the indicated references. The author instruction guide proposed by the journal was checked to adapt it to the requested format.

**The reviewer says:** The writing is generally clear and understandable, with good language quality. Tables and figures are mentioned in the text and add value by presenting results. The article complies with the ethical principles of research in human beings. However, there are some recommendations that I would like to make, with the aim of improving the manuscript. The names of families, orders and types of plants do not have to be written in italics.

**Our answer**: Observations raised regarding italic writing in plant types, orders, and families.

**The reviewer says:** Numbers greater than 10 should be written in letters and not in numbers, unless they are preceded by a unit of measurement recognized by the international system.

**Our answer**:

**The reviewer says:** With respect to scientific names, once the genus is named, the following times it appears, the initial of the genus is used, unless a paragraph begins with a scientific name.

 For example: Baccharis tricuneata (L.f.) Pers. (pichana) and Baccharis latifolia (Ruiz & Pav.) Pers. (chilca), should be changed to Baccharis tricuneata (L.f.) Pers. (pichana) and B. latifolia (Ruiz & Pav.) Pers. (chilca), among other examples.

**Our answer**: The observation on scientific names was lifted.

**The reviewer says:** In conclusion, the article has a very good quality in terms of structure, internal coherence, bibliographic support, and writing. With some minor adjustments in the reference format, the document could be ready to be indexed.

**Our answer**: Thank you for your final comments.
